# Sudanese emergency departments: a study to identify the barriers to a well-functioning triage

**DOI:** 10.1186/s12873-022-00580-1

**Published:** 2022-02-08

**Authors:** Bayan E. Ibrahim

**Affiliations:** Department of Community Medicine, Faculty of Medicine, University of Khartoum, Khartoum, Sudan

**Keywords:** Emergency department, Triage System, Barriers to effective triage

## Abstract

**Background:**

Triage system is a sorting system that categorizes patients on the basis of the severity of their condition and the availability of the resources in the emergency department. There has been little attention in the public literature to triage systems in Sudan. The aim of this study was to explore the triage system and identify the barriers in its application in hospitals in Sudan.

**Methods:**

A cross-sectional hospital based study was conducted at eight hospitals in Khartoum during December 2020. A multi-stage cluster sampling was applied. Data were obtained by interviewing emergency department staff using a structured questionnaire. The data were analyzed using statistical package for social sciences to find the association between various variables by chi-square test.

**Results:**

Most of the respondents stated that the triage system was deficient. Most of the participants of this study agreed that the role played by the administration in taking legislative decisions is crucial in improving the triage system. Among the factors found to be significant to a well-functioning triage system were, the need for substantial capital expenditure, p-value: 0.026, prudent legislative decisions, p-value: 0.026, adequate training of staff on means of performing efficient triaging, p-value: 0.007 and raising the awareness of the staff on the correct application of triage guidelines, p-value: 0.017.

**Conclusion:**

Currently there is no formal triage system in the State of Khartoum and has yet to be established. Policy making by administrators will play an important role in its implementation. It is suggested that prompt executive orders on improving the current triage system in Khartoum, should be carried out sooner than later, as the ripple effects of a well-functioning triage will decrease the average length of stay, mortality and morbidity rates and will eventually increase the patient’s satisfaction.

**Supplementary Information:**

The online version contains supplementary material available at 10.1186/s12873-022-00580-1.

## Background

Triage system is a sorting system that categorizes patients on the basis of the severity of their condition and the availability of the resources in the department; this guarantees that the quality and level of treatment rendered in the emergency room (ER) suits the acuity of patients' disease, acting as a basis for further initiation and management of patient evaluation, where patients with life threatening conditions are seen immediately; as seriously ill/injured patients are treated first, triage also speeds up the flow of the ER and increases family and patient satisfaction [1–3]. Upon the arrival of the patient into the Emergency Department (ED) the patient should be evaluated by a trained nurse. Triage is an abrupt assessment thus patients should not wait to be triaged [1]. Triage is the first point of contact when a person arrives at an ED, and the urgency of their condition is decided at this point [4].

Triage have been developed and introduced in several countries since the early 1990s. The seminal work of FitzGerald impacted the development of triage scales in some countries in the 1990s and 2000s being designed as 5-level scales, of these, the Australian Triage Scale, Canadian Emergency Department Triage and Acuity Scale, Manchester Triage Scale, and Emergency Severity Index have had the greatest influence on modern ED triage [5–8].

In high-income nations, triage has been widely implemented at EDs. While several triage tools have been developed, only a few have been specifically designed for resource-constrained areas. The World Health Organization (WHO) reports that the development of robust and efficient emergency care services could prevent a large proportion of deaths and injuries in low- and middle-income countries (LMIC). In most LMICs, where demand for emergency treatment far outstrips the material and human resources available, initial triage and emergency care provision are poorly handled. It is therefore critical that EDs have, depending on the urgency of their situation, frameworks for prioritizing patients [9–11].

The South African Triage Scale (SATS), a four-tiered acuity scale, was one of the few triage systems that had been systematically implemented and tested in developing countries [12, 13]. In South Africa, the SATS has proven to be a reliable and valid means of assessing undifferentiated ill and injured individuals, and it has been validated in other African and Asian countries [13, 14]. Another tool developed for emergency care in resource limited settings is the Interagency Integrated Triage Tool (IITT). It had been first launched for the COVID-19 pandemic as one of WHO's recommended tools. At the point of entry, the IITT is configured for use at ED. It groups patients into one of three color coded classes based on certain symptoms and indications (categories 1 and 2) or the absence of dangerous vital signs (category three) [15, 16].

The Federal Ministry of Health incorporated triage-based emergency care in the country's three largest hospitals (Khartoum Teaching Hospital, Khartoum North Teaching Hospital and Omdurman Teaching Hospital) in 2001 as a response to the increased morbidity and mortality found in non-triaged patients. In this system, patients are initially assessed by the nurse who conducts the primary triage and are then assigned to the required level of treatment [17]. Prior to the introduction of the triage system, all emergency and non-urgent patients were seen directly by junior doctors (house officers) in the same clinic [17].

In 2002, the Khartoum North Hospital created Sudan's first emergency care model. This model operates on the basis of triaging patients into one of three categories: special care, urgent care, and non-emergency care. Basic infrastructural modifications were made in both Omdurman and Khartoum Hospitals in 2005 to begin the new triage-based Emergency Departments. The patient was triaged into either the resuscitation area, area for critical ill patients or area for less severe cases [17].

Despite the fact that the triage system has been established in the three biggest hospitals in Khartoum State, there’s a general opinion that there is no formal triage system in Sudan [17]. The aim of this study was to explore the triage system and identify the barriers in its application and improvement in hospitals in Sudan.

## Methods and materials

This cross-sectional study conducted to explore the triage system and identify the barriers in its application was carried out at EDs of eight public hospitals dispersed across the seven localities of Khartoum State-Republic of Sudan during December 2020. Ibrahim Malik and Al-Tamayouz Teaching Hospitals both locate at Khartoum locality, Al-Nau Teaching Hospital locates at Karrari locality, Khartoum North Teaching Hospital locates at Bahri, Omdurman Teaching Hospital at Omdurman, Alban Jadeed Teaching Hospital at El-Haj Yousif, Turkish Teaching Hospital in Jabal-Awleya and Ombada Teaching Hospital at Ombada locality. The above hospitals were chosen by simple random sampling from all around Khartoum state, therefore, it is considered representative for the study in the state overall.

### Participants

Multi-stage cluster sampling was carried out with a sample size of 200 participants calculated using Slovin’s formula. Study sampling was done in two stages, first, hospitals were selected using simple randomization sampling then, using a cluster size of 25 from each hospital, data were collected from the ED staff. All doctors, including, house officers, medical officers, registrars, specialists, consultants, nurses as well as matrons and receptionists working in the EDs in the assigned hospitals, were included in the study.

A total of 56 nurses, 53 medical officers, 42 house officers, 19 receptionists, 6 registrars, 4 matrons, 3 specialists and 2 consultants that participated in the study (response rate=92.5%) were interviewed.

### Instrument

A written informed consent was attained. Data were obtained through a structured questionnaire by a trained research assistant. The questionnaire was translated into Arabic. A pilot survey was conducted to test for the questionnaire’s practicality and acceptability, correct tools’ errors and check for sampling procedures acceptability.

The questionnaire was designed using existing guidelines [5–8, 12] for triage. It contained 17 close-ended questions and it consisted of 3 sections: Sociodemographic attributes, assessment of the current triage system (if present) and the perceived barriers to the application or improvement of the triage system.

The sociodemographic attributes included the gender, age, job qualification and residence. Questions assessing the triage system were composed of participants responses about whether they acknowledged the presence of a triage system in the hospital, they are working at or not and for those that answered yes, their responses regarding whether they perceive that the system is well-functioning or not, the assessment of whether the patient is triaged immediately upon arrival, triage assessment time, if they were aware of the presence of a triage scale or any guidelines and if present if they were adhering to the given guidelines, whether the documentation and reporting of cases was proper and the safety during triage.

The section that explored the perceived barriers and ways to improve the system was a multiple answer question where participants were allowed to choose more than one option consisted of: the need for substantial capital expenditure and adequate provision of resources to the ED, the fundamental role played by the administration and legislative measures to be taken, adequate training of the staff on means of effective triage and raising the awareness amongst the staff on the importance of the correct application of triage guidelines.

### Data analysis and statistical methods

After the data was collected and entered on Microsoft Excel 2016. It was then verified and analyzed using IBM Statistical Package for Social Sciences software (version 23). The effect of several factors on staffs’ perception of whether the current triage system (if present) is well-functioning and efficient were studied. Sociodemographic attributes and participants’ responses from every hospital about the current triage system were all studied and analyzed. The effect of the perceived barriers by the participants on the their perspective of whether the triage system was effective or not was also studied.

Categorical data were summarised using frequency counts and proportions (%) and figures were generated. Continuous data were summarised using measures of central tendency, with mean and standard deviation. Cross tabulation was formulated. Chi-square analysis was conducted to reflect the association between the presence of an efficient and well-functioning triage and the aforementioned factors. Statistical significance was set as a p-value < 0.05.

Ethical approval was obtained from the Faculty of Medicine, University of Khartoum***.***

## Results

### Demographic data and participants’ qualifications

The study included 185 participants from 8 public hospitals. Almost equal number of participants were interviewed from each hospital. The mean age was 30.05 and with a standard deviation of 9.15 (ranging from 21–65 years). In regards to the participants’ job qualification, most were nurses, followed by medical officers, house officers, receptionists, registrars, matrons, specialists and consultants. The qualification of participants along with the rest of their demographics are shown in Table 1.

**Table 1:** Sociodemographic characteristics and qualification of participants (*n* = 185)

Item	Count	%
Gender		
Male	52	28.1%
Female	133	71.9%
Residence		
Khartoum	87	47.0%
Bahri	36	19.5%
Omdurman	60	32.4%
Other	2	1.1%
Qualification		
House Officer	42	22.7%
Medical Officer	53	28.6%
Registrar	6	3.2%
Specialist	3	1.6%
Consultant	2	1.1%
Nurse	56	30.3%
Matron	4	2.2%
Receptionist	19	10.3%

### ED triage system assessment

Out of the 185 participants, 57% acknowledged the presence of a triage system at their respective hospitals; however of those only 50% perceived it as being efficient and effective.

### Triage assessment

81.0% of the participants claimed that patients are immediately triaged upon their arrival to the EDs. 46.7% of the participants reported that the standard time for assessing the patient and assigning them to a category is 2–5 min and 62.7% stated that adequate documentation of the patient’s signs and clinical parameters takes place.

### Presence of triage guidelines

Out of the eight hospitals assessed in this study only two had an established triage protocol and a formal triage system namely, Ibrahim Malik Teaching Hospital and Omdurman Teaching Hospital which use the Sudan Triage Tool, a hospital specific tool developed in 2018, and IITT, respectively. At Ibrahim Malik Hospital the patient is triaged into one of three categories based on his vital signs into red where the patient requires immediate management within less than ten minutes, yellow meaning that the patient requires urgent management within 30 to 60 min, green indicating less urgent cases that can wait from 2 to 6 h or into black when the patient is pronounced dead.

In regards to the ED staff, 72.0% at Ibrahim Malik Teaching hospital and a mere 50.0% at Omdurman Teaching hospital were aware of the presence of a triage scale; of those, only 50.0% claimed to be adherent to the provided guidelines at the former hospital and 80.0% at the latter.

### Safety at Triage

A mere 19.5% stated that front-line staff of the ED receives training on minimization of aggression when dealing with patients or co-patients with challenging behaviors and only 21.6% reported that there are protocols and guidelines followed when dealing with such behaviors.

The results of participants’ responses from each hospital respectively, are all displayed in more details in an additional file [see Supplementary Table 1, Additional file 1].

### Perceived Barriers and Ways to Improve the Triage System

Most participants (78.4%) agreed that there is a fundamental role played by the administration and legislative measures to be taken for the improvement of the triage system followed by the need for substantial capital expenditure (62.7%) and adequate provision of resources to the ED, followed by, adequate training of the staff on means to performing an effective triage (48.6%) and raising the awareness amongst the staff on the importance of correct application of triage guidelines (28.1%).

The results of the chi-square test conducted to reflect the association between the participants’ perception of the presence of an efficient and well-functioning triage and the aforementioned factors are all shown in detail in an additional file [see Supplementary Table 2, Additional file 1]. The variables that had a significant association (*p*-value < 0.05) were:

1. The hospital they worked at. p-value: 0.007
2. Residence. p-value: 0.001
3. Immediate triage upon arrival time. p-value: 0.000
4. Substantial capital expenditure. p-value: 0.026
5. Administrative role. p-value: 0.026
6. Increasing staff’s awareness on correct application of guidelines. p-value: 0.017
7. Adequate Training on correct means of triage. p-value: 0.007
8. Minimization of Aggression Training. p-value: 0.000
9. Protocols for dealing with Aggressive Patients. p-value: 0.003

## Discussion

Triage is a relatively new concept in many African emergency centres. Although it had been broadly implemented across EDs in high income nations [5–8], it has yet to be systematically implemented and evaluated in LMICs for provision of quality emergency care. To the best of our knowledge this is the first study conducted to explore the triage system and identify barriers in its application in hospitals in Sudan. Our study demonstrates that Sudan lacks a formal and standardized triage system. This finding, is no surprise, as a previous study conducted back in 2001 involving seven developing countries revealed that 14 of 21 hospitals lacked an adequate system for emergency triage and also showed that methods for initial patient assessment led to delayed treatment [18, 19]. Our study determined the proportion of the assessed hospitals that had an established triage protocol in Khartoum State, the two hospital namely, Ibrahim Malik and Omdurman use the Sudan Triage Tool and IITT, respectively. Despite the fact that the evidence on triaging in LMICs is limited, the value of simple and context-specific approaches is widely recognized [16]. The key to improvement of the triage system is to reach a consensus on a single standardized system that allows nurses and other health care providers to use something that is simple yet consistent. This can be readily achieved by implementing an already developed and validated triage scale, an effective strategy that could be useful in ensuring uniform application of triage across EDs, as there is little evidence to back up the validity and reliability of the existing triage tools in LMICs [10]. The four-tier SATS is the most extensively researched of the small number of tools specifically designed for resource-limited settings [12, 16]. Although the tool has been shown to have adequate validity and reliability in a number of countries and contexts, it is also been noted to be too complicated for some settings, necessitating provider capacity that isn't always available [16].However, tools utilising three categories such as IITT are well-suited to developing EDs because they are intuitive and efficient [15, 16]. Furthermore, it fits to the three-level profile established by the Sudanese Ministry of Health in certain hospitals [17], and it would be a simpler system to teach to nurses and possibly nursing assistants in rural or understaffed smaller hospitals, since the IITT does not require calculation of a triage early warning score unlike the SATS. [12, 16]. Moreover, the training of the staff in a single specific triage system is essential to tackle the disparities in the perspectives of hospital staff on the existence of a well -functioning and efficient triage system at the hospitals they work at as demonstrated by a significant association (at a p-value of 0.007).

62.7% of our respondents agreed that there is a need for substantial capital expenditure and adequate provision of resources to the ED. This was coincident with the findings of a study conducted in Ghana with the purpose of assessing the capacity for care of emergency patients [20]. This capital should be invested in the standardization of the triage process and its implementation, particularly at the site of the triage station. The triage station should be accessible to the patients at their first point of contact with the health care provider in order to promote adherence to evidence-based guidelines [4]. It also should easily catch emergency medical service and ambulatory patients as they enter the ED. More so, basic equipment (such as personal protective equipment, a pulse oximeter, and so forth) should be available. In addition, the triage area should be spacious, ensure the privacy of the patient and the safety of the ED staff [8, 12]. Moreover, some of the capital should also be utilised in the training of the front line staff on dealing with challenging behavior on part of the patients and their relatives. Protocols should be implemented to provide a safe environment for the staff and patients which is crucial to ensure an efficient ER triage [6]. As demonstrated by an internal survey of emergency nurses and patient care assistants, violence is a continuing problem within the ED [21], and in another study one of the challenges related to emergency management was the inadequacy of the security section in the triage area [22]. Our study concluded a significant association between both pillars of the safety at triage area (minimisation-of-aggression training and protocols and procedures for dealing with challenging behaviour) and the staffs’ perception of a well-functioning triage (both had a p-value of 0.000 and 0.003, respectively). Interestingly, staff that described the triage as inefficient, also reported the lack of proper safety measures provided at the EDs.

Training ER staff on correct means of triage has the potential to improve efficiency and the delivery of emergency medical services and reducing overall mortality and morbidity. The importance of training can be exemplified by the results of our study where 48.6% of the participants asserted the necessity of adequately training the staff on means of performing an effective triage. Moreover, this study highlighted a gap on the knowledge on how triage is performed by several participants at their respective hospitals. Once more emphasizing on the importance of triage training. The closest we found to our results were those of several studies that emphasized the importance of training and educating the staff [2, 23, 24]. According to various triage guidelines it was found that patients should be immediately triaged upon arrival and the assessment time should take about 2 to 5 min as this is the most critical period spent in the ER [5–8, 12]. Fortunately, 81.0% of our staff reported that the patient is immediately triaged upon his arrival to the ED which is consistent with the findings of a study conducted in Sweden [25]. A significant association had been found between the staff perspective of assessment time (p-value of 0.000) and their perception of a well-functioning triage, similar to an audit conducted in Australia (p-value of 0.005) [4]. Overall, 46.7% of the participants reported that the triage assessment normally took 2 to 5 min. The lowest percentages among all the hospitals were 40.0% and 33.3%, these were obtained from Ibrahim Malik Hospital and Alban Jadeed Hospital respectively, where respondents reported that it takes place in the ideal time. In retrospect, the range of triage time was found to be 0.5–11.1 min in a study conducted at a trauma center [26]. The assessment of the adequacy of clinical care provided for the patient requires high quality of documentation. In addition, it can assist in producing evidence for legal purposes. However, nearly a third of our respondents stated that documentation is inadequate. Previous studies have indicated that missing data is common in emergency medicine [27]. Therefore, prior to adopting the triage role, the triage personnel should receive adequate training on triage prerequisites including proper documentation. This can be readily accomplished by enrolling the staff in the Médecins Sans Frontières (MSF) tembo training which offers an IITT educational course [28]. The staff that have received the MSF training can then serve as future triage trainers in Sudan.

Moreover, the respondents also emphazised on the cruciality of raising awareness of the ED staff on the correct means of application of the triage guidelines (28.1% of the responses). In the two hospitals with a triage scale, namely Ibrahim Malik Hospital and Omdurman hospital, only, 72.0% and 50.0% were aware of the presence of a scale in their hospitals, respectively. Only 50.0% from those were adherent to the guidelines in the former and 80.0% were adherent in the latter. Likewise, it was stated by a systemic review that triage wasn’t performed by the staff at all in some instances [9]. As for the rest of the hospitals in our study there was no objective triage or a triage scale in first place for the staff to follow which was also reported in a study conducted in Iran [21]. Thus, an efficient triage can be achieved by the improvement of the clinical competency of triage nurses, their encouragement to be motivated and the implementation of specific policies and instructions for the triage of patients.

The most important barrier to a well functioning triage reported by most of the participants of our study (78.4%) was the role played by the administration and legislative measures taken by authorities, as they agreed that proper administration is of essence in improving the triage system. Several studies described that improving access to emergency care in Africa calls for careful examination of the processes of governance. There is a need for legislation in order to provide a legal assurance of access to emergency services irrespective of the capacity to pay. The potential effect of legislative assurances of access to emergency care in Africa is demonstrated by constitutional and statutory rules and other governance frameworks [9–11, 24]. Therefore, the success of a triage demands undertaking a holistic integral approach in order to improve the system. The heart of this approach is a reformation in administration. Legal and infrastructure frameworks are required to approve the system’s operation and ensure that it is properly performed. In the context of LMICs, triage can necessitate additional equipment and space in the ER, hence requiring adequate resource allocation by administrations. Emergency administrators can also improve the quality of triaging patients by empowering triage nurses since they should have professional capabilities, including adequate knowledge about how to triage patients. These reforms are vital in improving patient survival and other health-related outcomes as well as gaining patient satisfaction. Moreover, it reduces overall health related expenditure. In addition, collaboration with the Ministry of Health is needed to ensure legislative measures are taken to improve the triage system. Measures that protect health care providers from violence and threats should be included in these legislations.

Further research is needed in this field once common standards of triaging have been established, primarily to answer questions about the impact, effectiveness and limitations of the triage system. Moreover, future studies will be required for evaluation of triage tools, impact of triage on waiting times, resource utilization, and patient satisfaction.

The study had certain limitations. One of which is the modest sample size of participants as data was not collected from 15/200 (7.50%) of ED staff who refused to participate in the study, which may have underpowered this study for the detection of additional associations. The methods were designed to identify an association, but not a direct causative effect, between the dependent and independent variables. The study was based on what participants viewed as hindrances to an effective triage, further studies are required for identifying specific causes. Other limitations included interviewer effect which was minimized by using a standard set of questions, as well as confounding bias associated with using a cross-sectional study design.

## Conclusion

Currently there is no formal triage system in the State of Khartoum and has yet to be established. Policy making by administrators will play an important role in implementing a well- functioning triage, as its implementation will be cost-effective and will require proper allocation of resources, which is lacking in low-income countries, including Sudan. Prompt executive orders on improving the current triage system in Khartoum should be carried out sooner than later, as the ripple effects of a well-functioning triage will decrease the average LOS, mortality and morbidity rates and will eventually increase the patient’s satisfaction.

## Supplementary Information

**Additional file 1: Supplementary Table 1.** Responses of participants regarding triage prerequisites at their respective hospitals (*n*=185). **Supplementary Table 2.** Pearson Chi-Square results for association between a well-functioning triage and various factors (*n*=185).

## Data Availability

The datasets generated and/or analysed during the current study are available in Figshare repository, [http://doi.org/10.6084/m9.figshare.19092065].

## Abbreviations

ER: Emergency Room
ED: Emergency Department
WHO: World Health Organisation
LMIC: Low- and Middle-Income Countries
SATS: South African Triage Scale
IITT: Interagency Integrated Triage Tool
MSF: Médecins Sans Frontières
