# Supplementary material for: Sudanese emergency departments: a study to identify the barriers to a well-functioning triage
Source: BMC Emerg Med. 2022 Feb 8;22:22. doi: 10.1186/s12873-022-00580-1 (PMC8822826; doi:10.1186/s12873-022-00580-1)
Supplement: Supplementary file 1 — Additional file 1: Supplementary Table 1. Responses of participants regarding triage prerequisites at their respective hospitals (n=185). Supplementary Table 2. Pearson Chi-Square results for association between a well-functioning triage and various factors (n=185). [file 12873_2022_580_MOESM1_ESM.docx]

Supplementary Table 1: Responses of Participants regarding triage Prerequisites and average length of stay at their respective hospitals (n=185)

|  | | Participants in Hospital‎ | | | | | | | |
| --- | --- | --- | --- | --- | --- | --- | --- | --- | --- |
|  |  | Ibrahim Malik | Al-Nau | Khartoum North | Omdurman | Al-Tamayouz | Alban Jadeed | Turkish | Ombada |
| Triage System Presence | Yes | 100.0% | 90.0% | 64.0% | 80.0% | 64.0% | 36.0% | 4.0% | 0.0% |
|  | No | 0.0% | 10.0% | 36.0% | 20.0% | 36.0% | 64.0% | 96.0% | 100.0% |
| Well-Functioning Triage | Yes | 36.0% | 77.8% | 25.0% | 65.0% | 31.3% | 66.7% | 100.0% | 0.0% |
|  | No | 64.0% | 22.2% | 75.0% | 35.0% | 68.8% | 33.3% | 0.0% | 0.0% |
| Immediate Triage upon Arrival | Yes | 88.0% | 88.9% | 68.8% | 90.0% | 75.0% | 55.6% | 100.0% | 0.0% |
|  | No | 12.0% | 11.1% | 25.0% | 5.0% | 25.0% | 11.1% | 0.0% | 0.0% |
|  | I don't know | 0.0% | 0.0% | 6.3% | 5.0% | 0.0% | 33.3% | 0.0% | 0.0% |
| Triage Assessment Time | <2 minutes | 16.0% | 16.7% | 0.0% | 15.0% | 12.5% | 11.1% | 0.0% | 0.0% |
|  | 2-5 minutes | 40.0% | 61.1% | 43.8% | 55.0% | 43.8% | 33.3% | 0.0% | 0.0% |
|  | >5 minutes | 36.0% | 0.0% | 18.8% | 20.0% | 37.5% | 11.1% | 100.0% | 0.0% |
|  | I don't know | 8.0% | 22.2% | 37.5% | 10.0% | 6.3% | 44.4% | 0.0% | 0.0% |
| Adequate Documentation and reporting of cases | Yes | 56.0% | 60.0% | 60.0% | 68.0% | 60.0% | 56.0% | 76.0% | 66.7% |
|  | No | 40.0% | 40.0% | 36.0% | 28.0% | 28.0% | 36.0% | 16.0% | 26.7% |
|  | I don't know | 4.0% | 0.0% | 4.0% | 4.0% | 12.0% | 8.0% | 8.0% | 6.7% |
| Minimization of Aggression Training | Yes | 12.0% | 20.0% | 8.0% | 40.0% | 4.0% | 24.0% | 28.0% | 20.0% |
|  | No | 84.0% | 80.0% | 88.0% | 48.0% | 76.0% | 60.0% | 60.0% | 80.0% |
|  | I don't know | 4.0% | 0.0% | 4.0% | 12.0% | 20.0% | 16.0% | 12.0% | 0.0% |
| Protocols for dealing with aggressive patients | Yes | 12.0% | 25.0% | 20.0% | 40.0% | 16.0% | 28.0% | 16.0% | 13.3% |
|  | No | 84.0% | 70.0% | 68.0% | 48.0% | 76.0% | 64.0% | 64.0% | 86.7% |
|  | I don't know | 4.0% | 5.0% | 12.0% | 12.0% | 8.0% | 8.0% | 20.0% | 0.0% |

Supplementary Table 2: Pearson Chi-Square results for association between a well-functioning triage and various factors (n=185)

|  | | Well-Functioning Triage | | Chi-Square Value | P-value |
| --- | --- | --- | --- | --- | --- |
|  |  | Yes | No |  |  |
| Hospital‎ | Ibrahim Malik | 36.00% | 64.00% | 16.533 | 0.007 |
|  | Al-Nau | 77.80% | 22.20% |  |  |
|  | Khartoum North | 25.00% | 75.00% |  |  |
|  | Omdurman | 65.00% | 35.00% |  |  |
|  | Al-Tamayouz | 31.30% | 68.80% |  |  |
|  | Alban Jadeed | 66.70% | 33.30% |  |  |
| Gender | Male | 40.70% | 59.30% | 1.222 | 0.29 |
|  | Female | 52.60% | 47.40% |  |  |
| Job Qualification | House Officer | 52.90% | 47.10% | 10.412 | 0.166 |
|  | Medical Officer | 38.50% | 61.50% |  |  |
|  | Registrar | 66.70% | 33.30% |  |  |
|  | Nurse | 63.30% | 36.70% |  |  |
|  | Receptionist | 63.60% | 36.40% |  |  |
|  | Other | 0.00% | 100.00% |  |  |
| Residence | Khartoum | 34.00% | 66.00% | 15.852 | 0.001 |
|  | Bahri | 36.80% | 63.20% |  |  |
|  | Omdurman | 75.70% | 24.30% |  |  |
|  | Other | 50.00% | 50.00% |  |  |
| Age Groups | <24 years | 44.40% | 55.60% | 6.285 | 0.615 |
|  | 25-29 years | 50.00% | 50.00% |  |  |
|  | 30-34 years | 63.60% | 36.40% |  |  |
|  | 35-39 years | 44.40% | 55.60% |  |  |
|  | 40-44 years | 75.00% | 25.00% |  |  |
|  | 45-49 years | 57.10% | 42.90% |  |  |
|  | >50 years | 0.00% | 100.00% |  |  |
| Immediate Triage upon Arrival | Yes | 58.80% | 41.20% | 15.706 | 0.000 |
|  | No | 6.70% | 93.30% |  |  |
|  | I don't know | 20.00% | 80.00% |  |  |
| Triage Assessment Time | <2 minutes | 46.20% | 53.80% | 5.678 | 0.128 |
|  | 2-5 minutes | 61.20% | 38.80% |  |  |
|  | >5 minutes | 33.30% | 66.70% |  |  |
|  | I don't know | 42.10% | 57.90% |  |  |
| Minimization of Aggression Training | Yes | 87.00% | 13.00% | 16.592 | 0.000 |
|  | No | 39.50% | 60.50% |  |  |
|  | I don't know | 33.30% | 66.70% |  |  |
| Protocols for dealing with aggressive patients | Yes | 77.80% | 22.20% | 11.607 | 0.003 |
|  | No | 39.70% | 60.30% |  |  |
|  | I don't know | 40.00% | 60.00% |  |  |
| Adequate documentation and reporting of cases | Yes | 58.20% | 41.80% | 5.664 | 0.059 |
|  | No | 33.30% | 66.70% |  |  |
|  | I don't know | 40.00% | 60.00% |  |  |
| Substantial capital expenditure | Yes | 42.70% | 57.30% | 4.938 | 0.026 |
|  | No | 66.70% | 33.30% |  |  |
| Administrative role | Yes | 42.70% | 57.30% | 4.938 | 0.026 |
|  | No | 66.70% | 33.30% |  |  |
| Increasing staff’s awareness on correct application of guidelines | Yes | 33.30% | 66.70% | 5.745 | 0.017 |
|  | No | 58.00% | 42.00% |  |  |
| Adequate training on triage on correct means of triage | Yes | 39.10% | 60.90% | 7.176 | 0.007 |
|  | No | 65.90% | 34.10% |  |  |
